# A Newly Developed Chemically Defined Serum-Free Medium Suitable for Human Primary Keratinocyte Culture and Tissue Engineering Applications

**DOI:** 10.3390/ijms24031821

**Published:** 2023-01-17

**Authors:** Sergio Cortez Ghio, Martin A. Barbier, Emilie J. Doucet, Imad Debbah, Meryem Safoine, Gaëtan Le-Bel, Andréanne Cartier, Emilie Jolibois, Amélie Morissette, Danielle Larouche, Julie Fradette, Sylvain L. Guérin, Alain Garnier, Lucie Germain

**Affiliations:** 1 The Tissue Engineering Laboratory (LOEX), a Université Laval’s Research Center, QC G1V 0A6, Canada; 2 Department of Surgery, Faculty of Medicine, Université Laval, Quebec, QC G1V 0A6, Canada; 3 Regenerative Medicine Division, CHU de Québec-Université Laval Research Centre, Quebec, QC G1J 1Z4, Canada; 4 Department of Chemical Engineering, Faculty of Sciences and Engineering, Université Laval, Quebec, QC G1V 0A6, Canada; 5 Department of Ophthalmology, Faculty of Medicine, Université Laval, Quebec, QC G1V 0A6, Canada

**Keywords:** cell culture, tissue engineering, defined medium, stem cells, skin

## Abstract

In our experience, keratinocytes cultured in feeder-free conditions and in commercially available defined and serum-free media cannot be as efficiently massively expanded as their counterparts grown in conventional bovine serum-containing medium, nor can they properly form a stratified epidermis in a skin substitute model. We thus tested a new chemically defined serum-free medium, which we developed for massive human primary keratinocyte expansion and skin substitute production. Our medium, named Surge Serum-Free Medium (Surge SFM), was developed to be used alongside a feeder layer. It supports the growth of keratinocytes freshly isolated from a skin biopsy and cryopreserved primary keratinocytes in cultured monolayers over multiple passages. We also show that keratin-19-positive epithelial stem cells are retained through serial passaging in Surge SFM cultures. Transcriptomic analyses suggest that gene expression is similar between keratinocytes cultured with either Surge SFM or the conventional serum-containing medium. Additionally, Surge SFM can be used to produce bilayered self-assembled skin substitutes histologically similar to those produced using serum-containing medium. Furthermore, these substitutes were grafted onto athymic mice and persisted for up to six months. In conclusion, our new chemically defined serum-free keratinocyte culture medium shows great promise for basic research and clinical applications.

## 1. Introduction

Whether to minimize the risks associated with some culture components or to maximize culture efficiency, the methods involved in the serial cultivation of primary human keratinocytes for clinical applications have undergone several changes since their introduction by Rheinwald and Green in the late 1970s [1,2,3,4]. Strides have been most notably made in xenogeneic component replacement: human recombinant epidermal growth factor (EGF) has supplanted mouse-derived EGF, allogeneic irradiated human fibroblast feeder layers (iHFL) can replace murine irradiated 3T3 feeder layers (i3T3FL), and cholera toxin can now be substituted by various molecules, such as isoproterenol, a synthetic and well-characterized cyclic AMP inducer [5,6].

To date, however, bovine serum (BS) is still widely used to produce tissue-engineered skin substitutes in a clinical setting [6,7,8,9]. Serum is the acellular portion of the blood and contains a plethora of factors, such as hormones, vitamins, transport proteins, attachment factors, growth factors, and trace elements. Although BS may be a key component in retaining the epithelial stem cells needed to ensure long-term skin substitute persistence after grafting [9], its composition is undefined and subject to seasonal and geographical variations [10,11]. This can ultimately interfere with experimental reproducibility and product consistency [12,13]. In addition, there are possible risks of BS being contaminated with prions and new or uncharacterized viruses [10,14]. BS supply is also highly dependent on the beef consumption industry, which is itself dependent on a multitude of environmental and regulatory factors [15,16]. Ultimately, this has led to concerns about fraudulent sera blending in response to the increasing BS demand in what appears to be a loosely regulated market [17,18].

Considering these limitations, efforts have been made towards the development and the commercialization of serum-free and defined culture media for the culture of keratinocytes [19]. Reports of keratinocytes grown in serum-free conditions in the literature [20,21,22,23] prompted us to conduct our own expansion experiments with a handful of commercially available serum-free media. We tested Gibco’s Defined Keratinocyte Serum-Free Medium (DK-SFM), as well as the optimized (KGM-2) and the completely defined (KGM-CD) versions of Lonza’s Keratinocyte Growth Medium (KGM) [24], prevalent serum-free media at the time [20,21,25]. In cultured monolayers, we observed morphological anomalies, decreased proliferative potential, and early growth arrest in human primary keratinocytes cultured with these three commercially available serum-free media [24] when compared with keratinocytes grown in conventional BS-containing complete keratinocyte culture medium in conjunction with a feeder layer [26]. Subsequent in-house tissue-engineered skin substitute (TES) experiments with CELLnTEC’s keratinocyte defined medium Cnt-07 and Lonza’s KGM-2 revealed that these media failed to support the formation of stratified and manipulable epidermal sheets (unpublished data). These results were in line with those of Lamb and Ambler, who tested Lonza’s KGM-Gold and Gibco’s EpiLife and who showed that they were both ineffective in supporting the formation of a stratified epidermis on their TES model without the addition of BS [27]. Other serum-free media that have been used to produce TESs for clinical applications are Lifeline’s Dermalife K [28] and Sigma-Aldrich’s MCDB 153 [23]. However, Lifeline’s advertised 15 population doublings are at least three times below what we can expect with serum-containing keratinocyte culture medium [24], and MCDB 153 must typically be supplemented with undefined bovine pituitary extract (BPE) [29]. Lonza’s KGM has also been used to produce TESs in a clinical setting, but only to expand the keratinocytes, after which BS was introduced into the system [30]. On the other hand, it has recently been demonstrated that, by using conventional BS-containing medium, autologous self-assembled skin substitutes (SASS) displaying a properly stratified neo-epidermis can be produced, grafted, and persist on severely burned patients [6].

This illustrates that there is a clinical need for a serum-free medium suitable for the expansion of human keratinocytes and the production of TESs while retaining epithelial stem cell populations [9]. The objective of this study was to evaluate the properties of keratinocyte cultures and TESs produced with the chemically defined Surge Serum-Free Medium (Surge SFM) before and after grafting on a murine model.

## 2. Results and Discussion

### 2.1. Surge SFM Supports the Growth of Freshly Isolated Human Skin Keratinocytes in Culture 

**Figure 1:**
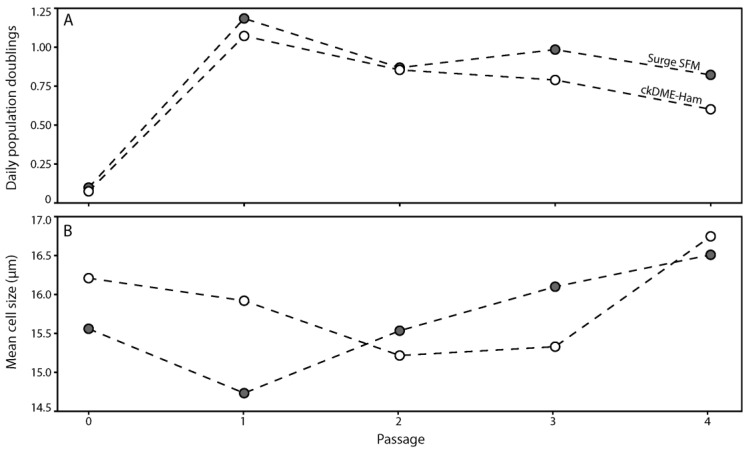
Line-connected dot plots of (**A**) the number of daily population doublings and (**B**) the mean cell size (μm) of k2 primary keratinocytes over five passages. Dot fill colors indicate the medium used to isolate and culture keratinocytes (gray: Surge SFM and white: ckDME-Ham). Dots are means of triplicates. Technical variability did not exceed 15%.

Because we initially developed Surge SFM using a single cryopreserved keratinocyte population (k1) cultured on an i3T3FL, we first wanted to determine if Surge SFM could efficiently support the growth of freshly isolated keratinocytes on an iHFL. We thus proceeded with the isolation and culture of human keratinocytes from an abdominal skin biopsy (k2) using either Surge SFM or ckDME-Ham for five passages. We calculated daily population doublings (Figure 1A) and measured the mean cell size (Figure 1B) for each passage (mean freshly isolated cell size prior to primary culture seeding was 11.55 μm). The k2 keratinocyte population doubled at an equivalent rate and displayed a similar mean cell size whether Surge SFM or ckDME-Ham was used. Note that cell size has been shown to be a good indicator of the level of commitment into differentiation for keratinocytes [31] and is usually inversely correlated to proliferation metrics [5], as is the case here. Additionally, and as expected, keratinocytes cultured in either medium displayed a cuboidal shape and formed colonies in monolayers cultured in the presence of iHFL. These data suggest that isolating and culturing human keratinocytes with either Surge SFM or ckDME-Ham yields comparable results in terms of proliferative potential and general aspects.

### 2.2. Surge SFM Supports the Growth of Cryopreserved Primary Human Keratinocytes in Culture

**Figure 2:**
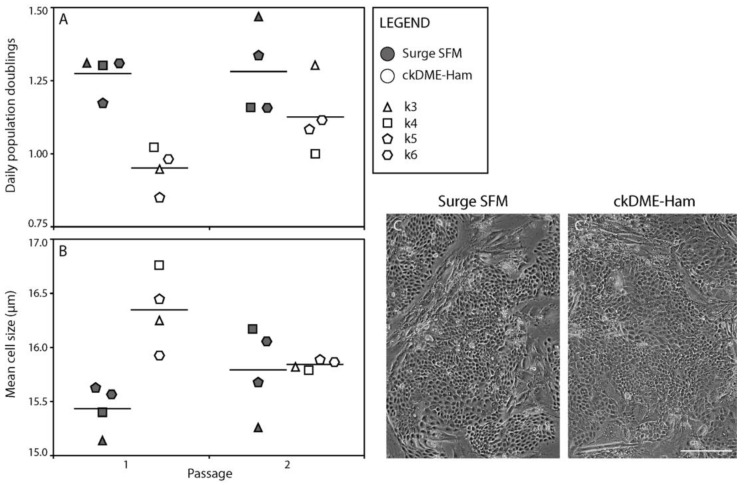
Dot plots of (**A**) the number of population doublings/day and (**B**) the mean cell size (μm) over two passages in culture of primary keratinocytes that were previously cryopreserved. Crossbars represent biological means. Dot fill colors indicate the medium used to culture keratinocytes (gray: Surge SFM and white: ckDME-Ham). Dot shapes identify keratinocyte populations (k3: triangle, k4: square, k5: pentagon, and k6: hexagon). Dots are means of triplicates. (**C**,**C’**) Phase contrast micrographs of k4 keratinocytes in P1 after 4 days of culture with either (**C**) Surge SFM or (**C’**) ckDME-Ham. Sharpness was increased 50% for visual clarity. Scale bar: 50 µm.

Next, we investigated if we could reproduce what we observed with k1 during the development of the Surge SFM medium, i.e., whether Surge SFM could be used to culture different cryopreserved keratinocyte populations that had been cultured with serum-containing ckDME-Ham on i3T3FL prior to being cryopreserved in P0. Cells isolated from different anatomical sites (k3, k4, k5, and k6) were thawed and cultured for two passages using either Surge SFM or ckDME-Ham. Once again, we calculated daily population doublings (Figure 2A) and measured the mean cell size (Figure 2B) of the keratinocytes for each passage. We found that daily population doublings for keratinocytes cultured with Surge SFM were increased by 0.32 when compared to their counterparts cultured with ckDME-Ham in P1 (*p* = 0.007). However, the difference of 0.15 daily doublings observed in P2 was not significant (*p* = 0.206). Similarly, we found that keratinocytes cultured in Surge SFM were smaller than those cultured with ckDME-Ham in P1 (0.91 μm smaller, *p* = 0.005), but not in P2 (*p* = 0.993). Keratinocytes cultured with Surge SFM formed colonies (Figure 2C) reminiscent of those with ckDME-Ham (Figure 2C’) and did not display morphological anomalies (Figure 2C). These data suggest that thawing and culturing keratinocytes with Surge SFM results in an increase in growth rate and that their morphology remains rather similar to that of cells cultured with ckDME-Ham, even if keratinocytes were grown with BS prior to cryopreservation. These results were very encouraging as sudden serum deprivation can induce apoptosis in keratinocytes [32,33] and other cell types [34]. Moreover, when compared with previous experiments [35,36], it was surprising that the newborn skin keratinocytes (k6) were similar to the adult skin keratinocytes. However, in the experiments that we describe herein, we only tested one cell population and cultured the cells for two passages only.

### 2.3. Surge SFM Supports the Retention of Epithelial Stem Cells in Culture

**Figure 3:**
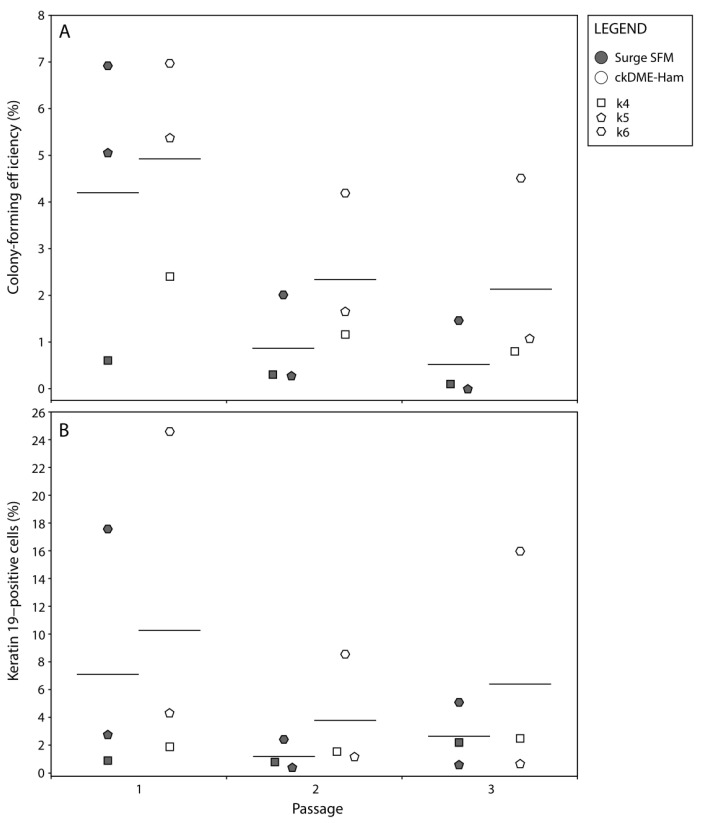
Dot plots of (**A**) keratinocytes’ holoclone (more than 4 mm in diameter) colony-forming efficiency (%) and (**B**) flow cytometry data for K19-positive cells (%) over three passages. Crossbars represent biological means. Dot fill colors indicate the medium used to culture keratinocytes (gray: Surge SFM and white: ckDME-Ham). Dot shapes identify keratinocyte populations (k4: square, k5: pentagon, and k6: hexagon). Dots are means of quintuplicates in (**A**) and means of triplicates in (**B**). Technical variability did not exceed 15%. Isotype controls did not exceed 0.1% K19-positive cells.

One of the major challenges of keratinocyte culture, most notably for clinical applications, is the retention of epithelial stem cells [9]. To ensure long-term graft persistence and regeneration, epithelial stem cells must be retained and carried through keratinocyte subcultures and TES production. ckDME-Ham has been shown to be effective in such regard for the treatment of burn patients with SASSs [6]. To determine if similar results could be achieved with Surge SFM, we carried out holoclone colony forming efficiency (CFE) and flow cytometry assays with k4, k5, and k6 keratinocytes in cultured monolayers over three passages. We found that the CFE of keratinocytes cultured with Surge SFM was decreased on average by 1.27 percentage points when compared to that of keratinocytes cultured with ckDME-Ham (*p* = 0.017; Figure 3A). Additionally, regardless of which medium was used, CFE was reduced by 3.02 percentage points on average in both P2 and P3 when compared with P1 (*p* < 0.001; Figure 3A). The decrease in CFE as the number of passages increases was expected [5], but the observed reduced clonogenicity of keratinocytes cultured with Surge SFM rather than with ckDME-Ham, although small, was surprising considering our population doublings and cell size results, presented in Figure 2.

We thus decided to verify these results with flow cytometry assays in which K19 was used as an epithelial stem cell marker [37,38,39]. K19-positive cells were detected in all samples. A representative example of the flow cytometry data is shown in Supplementary Figure S1. In line with the CFE data, there was a 3.17 percentage point decrease in K19-positive cells on average with Surge SFM when compared with ckDME-Ham. However, in contrast with previous results, this difference was not significant (*p* = 0.169; Figure 3B). Moreover, no passage-dependent statistically significant effects were detected (*p* = 0.101; Figure 3B). These slight discrepancies between CFE and flow cytometry assay data seem to be attributable to the fact that k5 breast skin keratinocytes exhibited rather high clonogenicity but encompassed relatively few K19-positive cells, especially in P1. Note that population variability was expected in these experiments because epithelial stem cell proportions vary depending on donor age [39]. The higher proportion of K19-positive cells in the keratinocyte population from newborn compared to adult populations is in agreement with the literature [35,39]. Taken together, these results indicate that although the observed clonogenicity is slightly decreased when culturing keratinocytes with Surge SFM rather than ckDME-Ham, cells presenting epithelial stem cell-like features can effectively be retained with Surge SFM.

### 2.4. Surge SFM’s Effect on Keratinocytes’ Transcriptome Is Minute but Could Promote Growth and Stifle Differentiation through EHF Regulation

**Figure 4:**
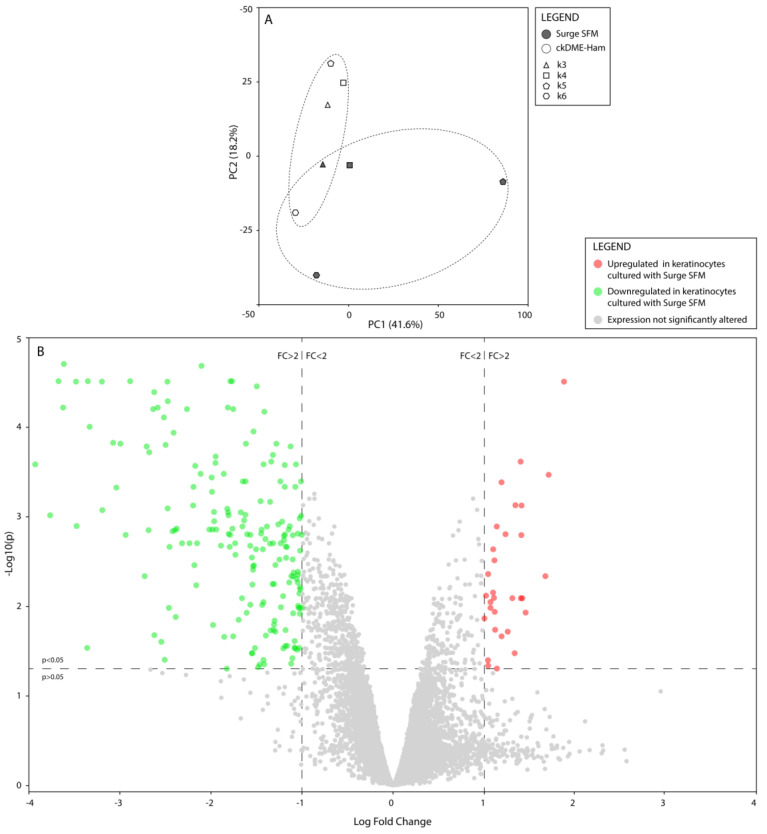
(**A**) Principal component analysis of normalized and filtered gene expression data. Dot fill colors indicate the medium used to culture keratinocytes (gray: Surge SFM and white: ckDME-Ham). Dot shapes identify keratinocyte populations (k3: triangle, k4: square, k5: pentagon, and k6: hexagon). Dotted ellipses represent both medium type clusters. (**B**) Volcano plot illustrating the results of the paired differential analysis of gene expression between keratinocytes cultured with Surge SFM and those cultured with ckDME-Ham. Dots represent individual genes. Gray genes’ expression was not significantly altered by culturing keratinocytes with Surge SFM rather than ckDME-Ham as opposed to colored genes whose expression was significantly (*p* < 0.05) modified by at least a 2-fold change in either direction (green: downregulated and red: upregulated).

To further assess how culturing keratinocytes with Surge SFM rather than ckDME-Ham would affect the cells, notably at the molecular level, we proceeded with transcriptomic profiling assays. We analyzed the transcriptomes of k3, k4, k5, and k6 keratinocytes cultured with either Surge SFM or ckDME-Ham. A principal component analysis revealed that most of the gene expression variance could be attributed to population divergence, notably between k5 and k6. Samples appeared to cluster relative to which culture medium was used, but clusters seemed to intersect (Figure 4A). This suggests that the gene expression profiles of keratinocytes grown in Surge SFM do not heavily differ from those of keratinocytes grown in ckDME-Ham. This was confirmed by our paired differential gene expression analysis, which revealed that, out of the approximate 60,000 hybridization targets on the microarray, only 220 genes (0.37%) were significantly (*p* < 0.05) differentially expressed (at least 2-fold change) between cells cultured in either medium (Supplementary Figure S2). Of these 220 genes, 189 (85.9%) were downregulated in keratinocytes cultured with Surge SFM when compared to keratinocytes cultured with ckDME-Ham, and 31 (14.1%) were upregulated (Figure 4B).

**Figure 5:**
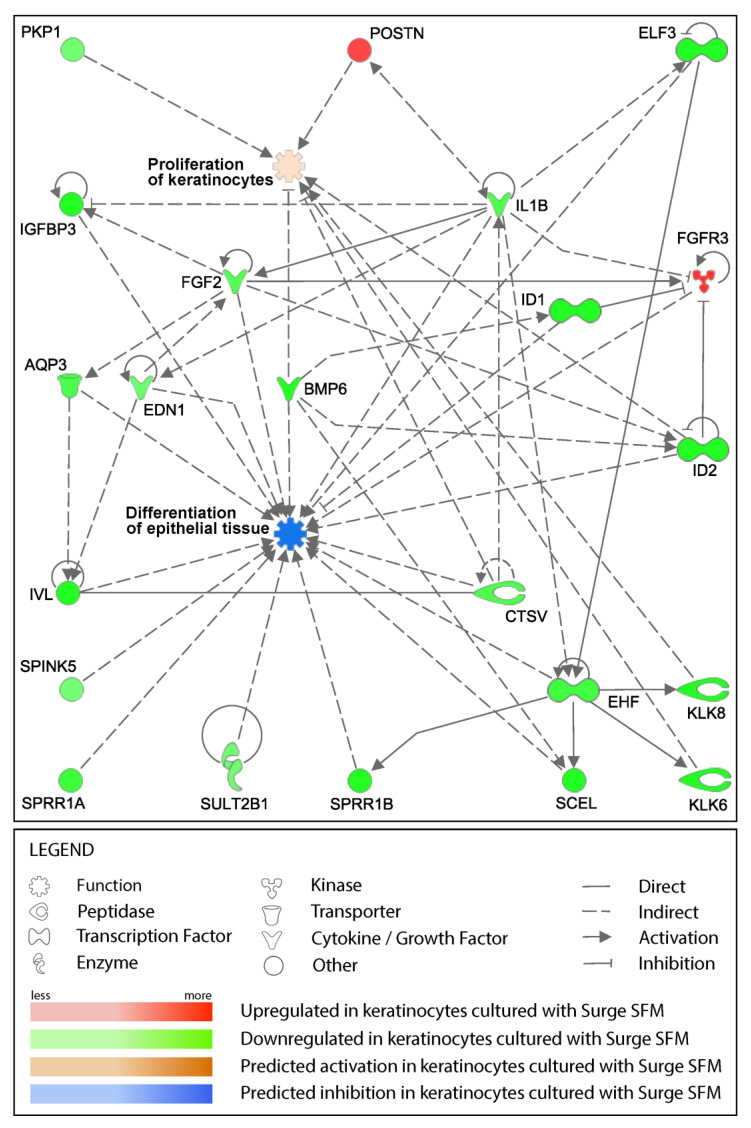
Gene–gene and gene–function interaction network built around the two biological functions of interest, “Proliferation of keratinocytes” and “Differentiation of epithelial tissue”, using the gene expression differential analysis data.

To investigate how Surge SFM could drive these gene expression differences and especially what roles these differentially expressed genes could play, we used the Ingenuity Pathway Analysis software to detect potential upstream regulators, build interaction networks, and make in silico biological function predictions. The upstream regulator analysis revealed that the decreased expression of the Ets homologous factor (EHF; LogFC = −1.53) in keratinocytes cultured with Surge SFM rather than with ckDME-Ham could explain the decreased expression of 18 other genes in our dataset (z-score = −2.86, *p* = 2.4 × 10^−24^). EHF is a member of the epithelium-specific Ets transcription factor subfamily, which has been shown to regulate over 400 genes involved in human keratinocyte differentiation and epidermis development [40]. We then proceeded to assemble gene–function interaction networks around biological functions of interest (Figure 5). Because we observed culture medium-related differences in the growth rate of the cryopreserved keratinocytes used for the microarray analysis, we selected “Proliferation of keratinocytes” as the first function of interest. Then, because EHF and the genes that it regulates have been associated with keratinocyte differentiation and epithelial tissue development, we added the “Differentiation of epithelial tissue” node to the network. Using our gene expression data and IPA’s “Molecule Activity Predictor” tool, we could determine in silico that keratinocyte proliferation would likely be increased and that epithelium differentiation would likely be decreased if Surge SFM was used instead of ckDME-Ham. Interestingly, the direction of the in silico prediction for proliferation matched our in vitro culture observations for these populations. On the other hand, IPA’s prediction on tissue differentiation was intriguing as a well-differentiated and stratified epithelium is essential in maintaining the epithelial barrier.

### 2.5. Surge SFM Supports the Formation of a Properly Stratified Neo-Epithelium on Tissue-Engineered Skin Substitutes

**Figure 6:**
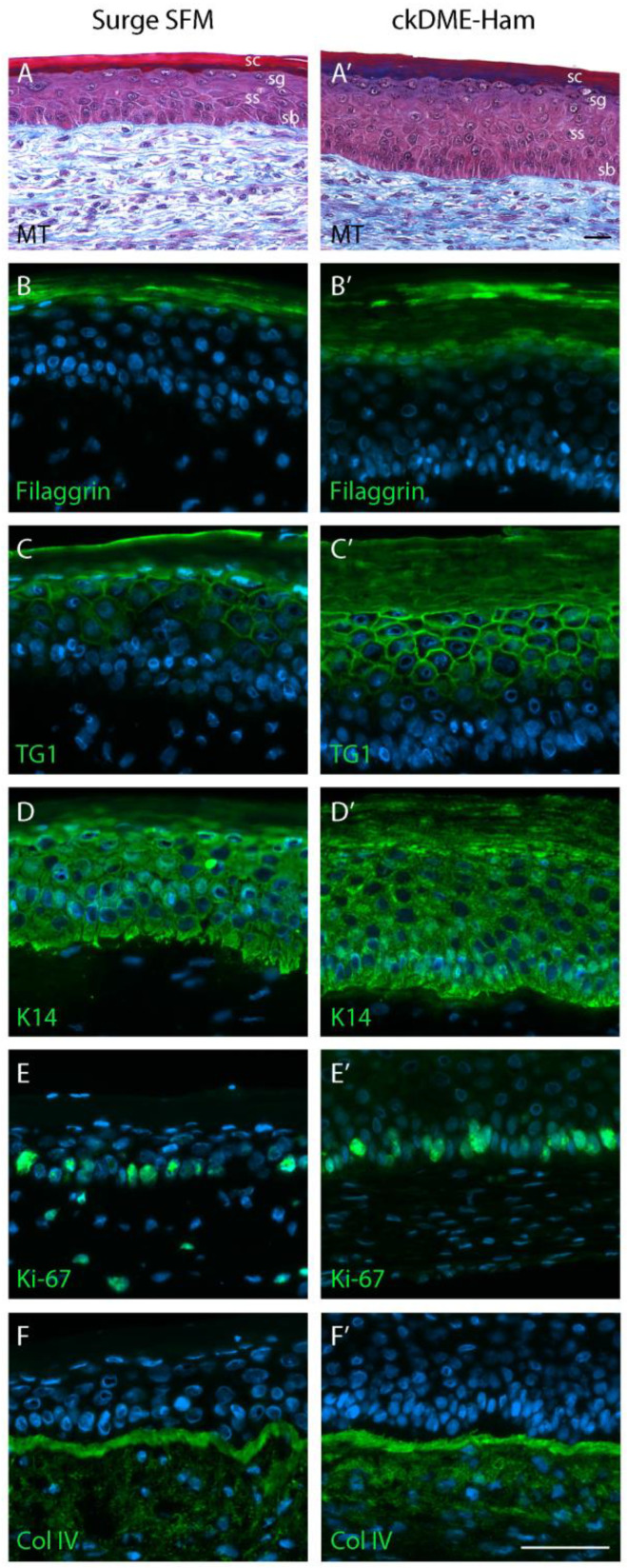
Histology and immunostaining of the TES produced with (**A**–**F**) Surge SFM or with (**A’**–**F’**) ckDME-Ham. Epidermal layers are identified (sb: *stratum basale*, ss: *stratum spinosum*, sg: *stratum granulosum*, sc: *stratum corneum*). (**A**,**A’**) Masson’s trichrome staining. Immunofluorescence staining against (**B**,**B’**) filaggrin, (**C**,**C’**) transglutaminase 1 (TG1), (**D**,**D’**) keratin 14 (K14), (**E**,**E’**) Ki-67, and (**F**,**F’**) type IV collagen (Col IV). Cell nuclei were stained with Hoechst reagent. Scale bars: 50 µm.

Designing a serum-free medium suitable for the proper stratification of the epithelial tissue in TESs was one of our main objectives for this study. Therefore, we continued with the production of TESs using either Surge SFM or ckDME-Ham for both the expansion (keratinocyte in cultured monolayers) and the subsequent 3D substitute production steps. TES histological staining revealed that the epidermis was well attached to the tissue-engineered dermis. The *stratum basale* was organized in the typical single cuboidal cell layer palisade fashion. Cell–cell junctions resulted in the formation of a cohesive epidermis. All epidermal cell layers were present—the *stratum spinosum*, *stratum granulosum*, and *stratum corneum*. However, TESs produced with Surge SFM displayed a thinner epidermis than those produced with ckDME-Ham (Figure 6), which is in line with IPA’s decreased “Differentiation of epithelial tissue” prediction. Most of the difference in epidermis thickness seemed to stem from the suprabasal layers that contained a reduced number of cells, although the *stratum spinosum*, *stratum granulosum*, and *stratum corneum* generally displayed good histological organization with either medium (Figure 6A,A’).

To identify other potential differences between TESs produced with either medium, we then stained against various molecular markers. As expected, the *stratum granulosum* marker filaggrin appeared to be expressed by more cells in TESs made with ckDME-Ham rather than in those produced with Surge SFM (Figure 6B,B’). In line with this finding and reminiscent of IPA’s prediction about epithelial tissue development, the late differentiation marker transglutaminase (TG1) also appeared to be expressed in a greater number of cells in ckDME-Ham TESs (Figure 6C,C’). This could indicate that substitute differentiation is less advanced in Surge SFM, although it is worth noting that immunofluorescence staining is not a quantitative assay. Moreover, TESs produced with Surge SFM contained keratinocytes at all differentiation stages, including enucleated cells forming the protective *stratum corneum*. For both TESs produced with Surge SFM and with ckDME-Ham, keratin 14 (K14) was strongly labeled in the basal layer and in the suprabasal layers of the epidermis (Figure 6D,D’). The proliferation marker Ki-67 was expressed in cells of the basal layer and in a comparable manner between both conditions (Figure 6E,E’). The presence of type IV collagen (Col IV), an essential component of the basement membrane structure, was also observed in both types of TESs in a continuous fashion at the dermo-epidermal junction (Figure 6F,F’). Slight epidermal thickness and differentiation level differences aside, these results suggest that Surge SFM substitutes are structurally similar to ckDME-Ham substitutes.

### 2.6. Self-Assembled Skin Substitutes Produced with Surge SFM Display Long-Term Graft Persistence on Athymic Mice 

**Figure 7:**
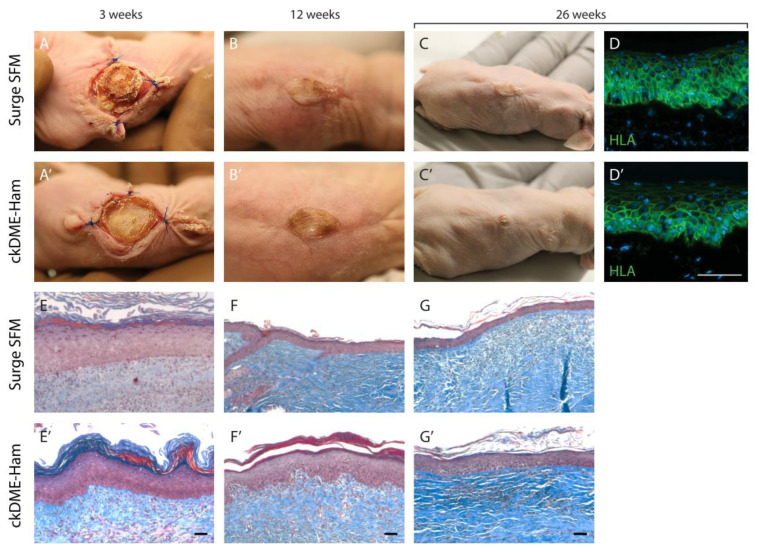
Functional analysis of human TESs produced with either (**A**–**G**) Surge SFM or (**A’**–**G’**) ckDME-Ham. TESs were grafted on athymic mice. Macroscopic view at (**A**,**A’**) three weeks, (**B**,**B’**) 12 weeks, and (**C**,**C’**) 26 weeks after grafting. (**D**,**D’**) Immunofluorescence staining against human leucocyte antigen (HLA) 26 weeks after grafting the TESs. Cell nuclei were stained with Hoechst reagent. Masson’s trichrome staining at (**E**,**E’**) three weeks, (**F**,**F’**) 12 weeks, and (**G**,**G’**) 26 weeks after grafting. Scale bar: 50 µm.

Lastly, functional studies were performed to evaluate whether the retained epithelial stem cells were sufficient to sustain the epithelial turnover within the neo-epidermis. TESs produced with either medium were grafted on athymic mice for up to 26 weeks (6 months). Figure 7 depicts a representative graft that persisted for the full duration of the experiment for TESs produced with each medium. Six out of seven grafts had taken in each group at postoperative day 7 (1 week). At postoperative day 21 (3 weeks), there were no significant observable differences between Surge SFM and ckDME-Ham grafts (Figure 7A,A’). At the 12-week mark, grafts had healed well (Figure 7B,B’). Mice were euthanized and graft tissue was sampled at different timepoints throughout the experiment. Two mice per condition were kept for the 26-week timepoint (Figure 7C,C’). Graft contraction was rather important but similar over time for both types of TESs. This is inherent to the rodent skin wound healing processes that rely on contraction mechanisms [41]. Moreover, post-graft matrix remodeling over time appears to be consistent with what is normally observed with serum-containing media (Figure 7E,E’,F,F’,G,G’). Lastly, in order to determine if the remaining tissue at the graft site 26 weeks after grafting originated from the initial human TES, immunolabeling of the human leukocyte antigen complex (HLA), a group of proteins encoded by the major histocompatibility complex (MHC) in humans, was performed. Epidermal cells were labeled (Figure 7D,D’), therefore indicating that they were indeed human cells. The presence of human cells at the graft site six months after transplantation is a suitable indicator of stem cell retention in the substitutes because these cells are necessary to ensure the survival and homeostasis (28-day epidermis turnover) of the neo-epidermis after grafting. These results strongly support that Surge SFM can be used to produce TESs suitable for grafting.

## 3. Materials and Methods

### 3.1. Cell Populations

All human keratinocyte populations studied (designated k1 to k6) were obtained from healthy participants undergoing aesthetic elective surgery. Keratinocytes were isolated from the excess skin of facelift (k1 and k4), lipectomy (k2 and k3), or breast reduction (k5) surgeries from 50-(k1), 58-(k2), 31-(k3), 56-(k4), and 37-year-old (k5) donors. Population k6 was isolated from foreskin tissue of a healthy neonate. Populations k1 and k6 were isolated from male donors, while k2 through k5 were isolated from female donors. Keratinocytes were either isolated and immediately subjected to serial cultivation for this study (k2) or were cryopreserved after primary culture (P0) for 5 to 13 years prior to their use in this study (k1, k3, k4, k5, and k6). A single fibroblast population (designated f7) was used for the TES experiments. It was isolated from the excess skin of a breast reduction surgery from an 18-year-old female donor and cryopreserved in P3 for 3 years prior to its use in this study.

### 3.2. Media

Our medium, Surge SFM, was compared to the conventional BS-containing complete keratinocyte culture medium (ckDME-Ham) that we use for routine keratinocyte culture [5,24,26,42]. The base medium (bDME-Ham) for both media was a three part Dulbecco’s modified Eagle’s medium (DMEM; Thermo Fisher Scientific, Ottawa, ON, Canada) and one part Ham’s F12 medium (Thermo Fisher Scientific) mixture supplemented with 3.07 mg/mL of NaHCO_3_ (J.T. Baker, Tekniscience, Terrebonne, QC, Canada) and 24.3 μg/mL of adenine (Sigma-Aldrich, St-Louis, MO, USA). ckDME-Ham additionally contained 5% *v/v* of inactivated FetalClone II serum (HyClone, Logan, UT, USA), which is a supplemented calf-serum-derived product, 5 μg/mL of insulin (Sigma-Aldrich), 10 ng/mL of EGF (Austral Biologicals, San Ramon, CA, USA), 0.4 μg/mL of hydrocortisone (Calbiochem, San Diego, CA, USA), 100 IU/mL of penicillin G (Sigma-Aldrich), 25 μg/mL of gentamicin (Galenova, Saint-Hyacinthe, QC, Canada), and 21.2 ng/mL of isoproterenol (Sigma-Aldrich). The final and complete formulation for Surge SFM is proprietary. A lighter version of Surge SFM containing a smaller number of additives was sometimes used (Surge SFM Light, i.e., to efficiently thaw or centrifuge keratinocytes in serum-free conditions).

Fibroblasts were cultured as previously described in fibroblast culture medium (fDME), which consisted of DMEM supplemented with 10% fetal bovine serum (FBS; Seradigm, Avantor/VWR, Montréal, QC, Canada), 100 IU/mL of penicillin G (Sigma-Aldrich), and 25 μg/mL of gentamicin (Galenova).

### 3.3. Keratinocyte Culture

Keratinocytes isolated for this study (k2) were processed as previously described [26]. Skin biopsies were transported from the operating room to the lab in transport medium on the same day that elective surgeries were performed. Specimens were then washed in antibiotics-containing PBS, cut into 3 mm × 100 to 150 mm strips, and incubated overnight at 4 °C in a 500 μg/mL thermolysin (Sigma-Aldrich)/4-(2-hydroxyethyl)-1-piperazineethanesulfonic acid (HEPES) solution [43]. The epithelium (epidermis and hair follicles) was subsequently peeled from the dermis with sterile tweezers. Epidermal strips were then incubated for 30 min in a trypsin/ethylenediaminetretraacetic acid (EDTA) solution (0.05% *w/v* of porcine trypsin 1–250 (Thermo Fisher Scientific), 0.01% *w/v* of EDTA (J.T. Baker), 2.8 mM of D-glucose (EMD Millipore, Burlington, MA, USA), 100 IU/mL of penicillin G, 25 μg/mL of gentamicin, and phenol red (J.T. Baker)) in PBS at 37 °C on a stirring plate. A 0.44 mg/mL soybean trypsin inhibitor (STI)/Surge SFM Light mixture was used to neutralize trypsin activity. Surge SFM Light was used to wash and resuspend the cell pellets. Keratinocytes were seeded at 1.4 × 10^4^ cells/cm^2^ on iHFL in either Surge SFM or ckDME-Ham. Cells were kept in incubators at 37 °C, 8% CO_2_, and 95 ± 5% humidity. Medium was changed three times per week. When cells attained 75–95% confluence, they were passaged using the above-mentioned trypsin/EDTA solution. Trypsin activity was neutralized by doubling the volume of the suspension with either the STI/Surge SFM Light mixture or ckDME-Ham. Keratinocytes were then centrifuged, resuspended, and seeded between 4 and 12 × 10^3^ cells/cm^2^ on iHFL in either Surge SFM or ckDME-Ham. When keratinocytes were cultured in triplicate, each flask was trypsinized individually. Proliferation and cell size data were acquired for each replicate. The flask with the total cell count closest to the mean of all replicates only was reseeded in triplicate for the following passage.

iHFL consisted of 60 Gy-irradiated human fibroblasts plated at 8 × 10^3^ cells/cm^2^ and cultured in either Surge SFM or ckDME-Ham for at least six (and up to 30) days before keratinocytes were seeded on top of them. Coating the flasks overnight with 5 μg/mL human plasma-derived fibronectin (FUJIFILM Irvine Scientific, Santa Ana, CA, USA) solubilized in sterile phosphate-buffered saline (PBS) was necessary for cells to adhere to the plastic when using Surge SFM, but not when using ckDME-Ham (as native BS fibronectin is present in ckDME-Ham at an estimated 1 μg/mL [44]). Medium was changed every seven days until keratinocytes were seeded. If keratinocytes were to be seeded on the same day, a medium change was scheduled; only half of the medium was changed so as to keep it partly conditioned by the iHFL.

### 3.4. Cryopreserved Cells

Cells thawed for this study (k1, k3, k4, k5, k6, and f7) were cryopreserved several years prior to the experiments described herein. Some cell isolation and culture methods thus differ from those employed for k2. Notably, for primary culture, ckDME-Ham contained cholera toxin at 10^−10^ M instead of isoproterenol, and keratinocytes were seeded at 2.8 × 10^4^ cells/cm^2^ on i3T3FL instead of 1.4 × 10^4^ cells/cm^2^ on iHFL.

i3T3FL consisted of 60 Gy-irradiated commercially available Swiss murine embryonic fibroblasts that were seeded simultaneously with freshly isolated keratinocytes at 2 × 10^4^ cells/cm^2^ in ckDME-Ham.

Fibroblasts (f7) were isolated by incubating the thermolysin-separated dermal strips overnight in a 0.125 U/mL collagenase H (Roche Diagnostics, Laval, Canada) solution at 37 °C on a stirring plate. Collagenase activity was then suppressed by doubling the volume of the suspension with fDME. Cells were subsequently centrifuged, resuspended, and seeded at 6.7 × 10^3^ cells/cm^2^ in fDME. Fibroblasts were subcultured when 100% confluence was reached [45].

Cells were cryopreserved by resuspending them in inactivated fetal calf serum (HyClone) containing 10% *v/v* of dimethyl sulfoxide (DMSO; Sigma-Aldrich) in cryovials (Thermo Fisher Scientific) on ice. Temperature was then slowly decreased by placing the cryovials in an isopropyl-alcohol-filled freezing container (Thermo Fisher Scientific) and storing them in a −80 °C freezer overnight. Cryovials were then stored in liquid nitrogen containers.

Cells were thawed by placing the cryovials in a 37 °C water bath for no more than one minute. The cell suspension was then diluted in 4 °C culture medium (either Surge SFM Light for keratinocytes or fDME for fibroblasts) at a 1:9 ratio, centrifuged, and resuspended in Surge SFM, ckDME-Ham, or fDME to be seeded. Keratinocytes were always reseeded on iHLF after thawing and at each passage. Note that keratinocytes from different cryovials were pooled during thawing and then split into both media so as to account for vial variability. Mortality rates were estimated with trypan blue staining and never exceeded 8%.

### 3.5. Serum-Free Medium Development

Briefly, Surge SFM was developed by screening several candidate factors identified as human keratinocyte growth and survival promoters from the available scientific literature with numerous successive two-level fractional factorial DOE [46]. bDME-Ham supplemented with antibiotics was used as a base medium to test the candidate factors. Based on proliferation and morphology alone, and relative to controls, underperforming factors were eliminated from round to round. A four-factor, three-level Box–Behnken experimental design was finally used to optimize the concentration of the most effective factors until a satisfactory formulation was obtained. These early development steps were conducted using only k1 in P2 on i3T3FL in 24-well plates so as to increase the testing throughput. ckDME-Ham was used as a positive control and antibiotic-containing bDME-Ham was used as a negative control.

### 3.6. Population Doublings

Keratinocyte population daily doublings (*Ddoubs*) were calculated as follows:

$$Ddoubs=\left(\;\frac{\log(Ktryp/Kseed)}{\log\left(2\right)}\;\right)/D$$

where *Ktryp* is the number of keratinocytes collected at the end of a given passage (measured with a Beckman Coulter automated cell counter following trypsinization), *Kseed* is the number of keratinocytes seeded at the beginning of the passage, and *D* is the length of the passage in days (from seeding to trypsinization and rounded to the quarter hour).

### 3.7. Colony-Forming Efficiency

Keratinocyte colony-forming efficiency assays were performed over 11 days in 25 cm^2^ culture flasks in quintuplicate on iHFL. Forty keratinocytes/cm^2^ were seeded. Medium was changed on days 4, 7, and 9. Colonies were rinsed with PBS, fixed with 3.7% formol (ACP Chemicals, Saint-Léonard, QC, Canada), and stained with a 0.1% *v/v* rhodamine solution (Sigma-Aldrich). Colony-forming efficiency (CFE) percentages were calculated as follows:

$$CFE=\frac{Nholo}{Kseed}\times 100$$

where *Nholo* is the number of colonies with a diameter larger than 4 mm (holoclones) [47], and *Kseed* is equal to 1000, which is the number of keratinocytes seeded into the culture flask for the assay.

### 3.8. Flow Cytometry Analysis

Keratinocytes were washed with PBS and incubated on ice for 30 min with 100 µL/million cells of 0.55 µg/mL Fixable Viability Staining 780 (FVS780; BD Biosciences, Franklin Lakes, NJ, USA) in PBS. Cells were then rinsed with PBS supplemented with 2% *v/v* of inactivated FetalClone II serum (HyClone) and 0.5 mM EDTA. They were then fixed for 45 min on ice with 1 mL/million cells of 3.7% *v/v* formaldehyde. Cells were kept at 4 °C no longer than three weeks prior to immunolabeling. Fixed cells were incubated for 5 min with a saponin-containing permeabilization solution (BD Perm/Wash; BD Biosciences), which was used throughout the immunolabelling procedure for both washing and antibody incubation steps. Cells were incubated at room temperature for one hour with a mouse anti-human keratin 19 (K19) clone A53-B/A2 [48] (gift from U. Karsten, Institute of Biological Sciences, University of Rostock, Germany) or its isotypic control (Agilent Technologies, Santa Clara, CA, USA). Following washes, cells were incubated for one hour in the dark with a goat anti-mouse phycoerythrin-conjugated IgG2a antibody (Thermo Fisher Scientific). Samples were analyzed with a fluorescence-activated cell sorter (BD FACSMelody; BD Biosciences), and 20,000 events were acquired. Only single cells and FVS780-negative cells were considered in the analysis. Gates were placed based on isotypic controls’ fluorescence. Data were analyzed using the FlowJo (v10.7; BD Bioscience) software [49].

### 3.9. Microarray Assay and Gene Expression Analyses

Using the RNeasy Mini Kit (QIAGEN, Hilden, Germany), total RNA was extracted from nearly 100% confluent k3, k4, k5, and k6 keratinocytes trypsinized in P2 (1 million cells per sample), centrifuged, snap-frozen in dry pellet form, and stored at −80 °C. RNA quality was assessed using an RNA 6000 Nano Kit (Agilent Technologies) and a 2100 Bioanalyzer Instrument (Agilent Technologies). A SurePrint G3 Human Gene Expression Microarray Chip (Agilent Technologies) in combination with the ArrayStar V12 software (DNASTAR, Madison, WI, USA) was used to generate linear gene expression data. All microarray data presented in this study comply with the Minimum Information About a Microarray Experiment (MIAME) requirements (GEO# GSE183594).

The ArrayStar microarray linear expression data for all four keratinocyte populations grown in either ckDME-Ham or Surge SFM were uploaded into Network Analyst [50], an R-based web tool, through which they were normalized using the variance stabilizing normalization method, and filtered to exclude low-abundance and low-variance genes (5th and 15th percentile, respectively). A principal component analysis was subsequently conducted with Network Analyst to examine how keratinocyte samples clustered. A pairwise differential gene expression analysis was then carried out between Surge SFM- and ckDME-Ham-grown keratinocyte samples using the limma statistical method, which resulted in a list of statistically differentially expressed genes (adjusted *p* < 0.05 and LogFC > 1.0). This list, including expression difference direction and intensity, was then uploaded into the Ingenuity Pathway Analysis (IPA; QIAGEN) software [51]. IPA was used to identify potential upstream regulators [51] and to compute gene interaction networks built around cellular functions of interest. To achieve this, we used permissive settings to connect (edge) every differentially expressed gene (node) and every potential biological function of interest (node) in such a way as to obtain both gene–gene and gene–function edges. Edgeless nodes were then removed. The in silico prediction tools of IPA were subsequently used to examine how the differentially expressed genes between cells grown in either medium would affect these functions. If the number of edges was insufficient to make a prediction for a given function of interest, that node was removed.

### 3.10. Skin Substitute Production

Keratinocyte population k5 and fibroblast population f7 were used to produce TESs. TESs were mostly produced as previously described, notwithstanding the use of Surge SFM [6,42]. Briefly, fibroblasts were thawed and subcultured for one passage. They were then trypsinized and reseeded in 85 cm^2^ NuncTM OmnitrayTM tissue culture plates with removable lids at 5.8 × 10^3^ cells/cm^2^ in fDME containing 50 μg/mL of ascorbic acid (Sigma-Aldrich) to promote extracellular matrix production and deposition. These cultures formed fibroblast-derived tissue sheets. Medium was changed three times per week for up to 22 days. In parallel, keratinocytes were thawed and subcultured for one passage on iHFL in either Surge SFM or ckDME-Ham. When they reached 75–95% confluence, keratinocytes were trypsinized and reseeded directly on top of one of each of three 18-day fibroblast-derived sheets at 10.6 × 10^4^ cells/cm^2^ in either Surge SFM or ckDME-Ham. Medium contained 50 μg/mL of ascorbic acid and was changed once per day for four days. The day after the keratinocytes were seeded, custom-made Ahlstrom grade 237 filter paper frames (inner dimensions: 46 × 76 mm, outer dimensions: 62 × 99 mm) were placed on the tissue sheets comprising both fibroblasts and keratinocytes. The frames were held in place with small sterile metal ingots. Three days later, medium was removed and the thin strips of tissue surrounding the frames were folded over the frames using sterile dissection forceps. Each framed tissue sheet could thus be slowly lifted from the culture plate and stacked onto a 22-day fibroblast-derived sheet. Surrounding tissue was once again folded over the paper frame and this process was repeated an additional time. The three-sheet constructs were then fixed to the frame with LIGACLIPS^®^ (Ethicon Endo-Surgery, Cincinnati, OH, USA). Substitutes were then placed on polypropylene membranes (Spectrum Labs, Rancho Dominguez, CA, USA) laid on a custom-made acrylic support to hold them at the air–liquid interface. The substitutes were further cultured for 10 days in the same media but EGF was not added at this stage. In total, eight TESs were produced: four with Surge SFM and four with ckDME-Ham. Each 45 × 75 mm TES was split into two 45 × 30 mm parts, which were grafted on nude mice, and one 45 × 15 mm strip, which was used for histological and immunofluorescence analyses.

### 3.11. Skin Substitute Grafting on Nude Mice

Fourteen 56-day-old athymic (Crl:CD1-Foxn1n; Charles River Laboratories, Laval, QC, Canada) female mice (mean weight of 25.07 g) were randomly split into two groups of seven mice and used as graft recipients for either Surge SFM or ckDME-Ham TESs. Mice were maintained under sterile housing conditions and received 30.75 mg/kg of subcutaneous ceftazidime (Teligent Inc., Buena, CA, USA) injections prior to grafting as well as 24 and 48 h after surgery. Before surgery, mice were administered a single 1 mg/kg dose of subcutaneous buprenorphine-SR (sustained release; CHIRON Compounding Pharmacy Inc., Guelph, ON, Canada) for analgesia. For the procedure, mice were anesthetized by 1.5–3% isoflurane (Baxter, Mississauga, ON, Canada) inhalation. They were also administered 20 mg/kg of carprofen (Zoetis Canada Inc., Kirkland, QC, Canada) preoperatively. Saline was subcutaneously administered daily to prevent dehydration for the first three postoperative days. To reduce mice skin contraction and graft tampering by the mice, medical-grade silicone Fusenig’s chambers (inner diameter of 2.5 cm) made in-house were used as previously described [52]. Briefly, a 2.5 cm^2^ circular piece of full-thickness skin was excised on the mid-back of each mouse and the base of a Fusenig’s chamber (outside diameter of 3.5 cm) was inserted into the opening. The mouse’s skin was attached over the base of the chamber with four to six polypropylene stitches (Prolene 4-0; Ethicon Inc., Cincinnati, OH, USA). To promote graft vascularization, the *panniculus carnosus* was removed, after which a single 45 × 30 mm TES was grafted directly on the exposed muscle of each mouse within the Fusenig’s chamber. Fusenig’s chambers were removed 3 weeks after grafting. Mice were euthanized 3, 12, and 26 weeks after grafting for tissue collection and analysis.

### 3.12. Histological and Immunofluorescence Analyses

TES tissue biopsies harvested before and after grafting were fixed overnight in HistoChoice^®^ (Amresco, Solon, OH, USA) and embedded in paraffin. Sections were then stained with Masson’s trichrome.

Biopsies of TES were also embedded in Tissue-Tek optimal cutting temperature compound (Sakura Finetek, Torrance, CA, USA), frozen in liquid nitrogen, and stored at −80 °C. Immunofluorescence assays were carried out on 5-µm-thick cryosections fixed with acetone (10 min at −20 °C). Sections were washed with PBS and then incubated with a blocking buffer (1% *w/v* of BSA in PBS) for 30 min. Following PBS rinses, tissue sections were incubated for 45 min with primary antibodies diluted in 1% *w/v* of BSA in PBS. The following primary antibodies were used: mouse monoclonal anti-Ki67 1:200 (BD Biosciences), rabbit anti-keratin 14 1:800 (K14; Cedarlane, Burlington, ON, Canada), rabbit anti-type IV collagen 1:400 (COL IV; Abcam), rabbit anti-transglutaminase 1 (TG1) 1:200 (ProteinTech, Rosemont, IL, USA), mouse anti-filaggrin 1:800 (Santa Cruz Biotechnology, Dallas, TX, USA), and anti-human leukocyte antigen A, B, C 1:25 (HLA-ABC; Biolegend, San Diego, CA, USA). Following PBS rinses, cells were incubated for 30 min with the following secondary antibodies: Alexa-488-conjugated goat anti-mouse antibody 1:800 (Invitrogen, Carlsbad, CA, USA) or donkey anti-rabbit 1:800 (Invitrogen). Cell nuclei were stained with Hoechst reagent (Sigma-Aldrich). For negative controls, primary antibody was either omitted or replaced with an isotypic negative control antibody.

### 3.13. Statistical Analyses

Statistical analyses were carried out using R (v3.6.1, R Studio v1.1.456) [53]. Repeated-measures linear models were fit using the nlme package (v3.1-140) [54]. When significant (*p* < 0.05) effects were detected, Tukey post-hoc tests were performed with the multcomp package (v1.4-13) [55] for single-factor effects and the emmeans package (v1.3.5.1) [56] for interaction effects; significant (*p* < 0.05) pair-wise comparisons were reported (estimated effect size and *p* value). Homoskedasticity and normality of residuals were verified with residuals over fitted value and QQ plots, respectively.

## 4. Conclusions

Our objective was to develop a chemically defined serum-free medium that would support human primary keratinocyte cultures and TESs phenotypically equivalent to those successfully produced with the conventional serum-containing medium, ckDME-Ham. To achieve this, we used a statistical DOE approach to screen a large number of candidate factors and efficiently formulated our new medium, Surge SFM.

Surge SFM’s formulation is advantageous. Using Surge SFM does not require significant culture protocol changes other than coating the flasks with fibronectin and using trypsin inhibitors. Furthermore, Surge SFM could become xenogen-free by replacing the BSA that it contains with purified human albumin. In addition, and as opposed to many of the commercially available keratinocyte serum-free media, Surge SFM was developed to culture keratinocytes alongside a feeder layer. The latter has been shown to increase keratinocytes’ growth rate and clonogenicity, as well as promote the retention of epithelial stem cells [24,57]. In contrast with other commercial media that are designed for feeder-free systems, Surge SFM was formulated as a high-calcium medium, similarly to ckDME-Ham, to favor a proper epithelium structure. Calcium is required to form desmosomes and cell junctions between keratinocytes. It thus plays an important role in cell–cell attachment during differentiation and is likely to contribute to the adequate neo-epidermal stratification observed with Surge SFM [58,59].

Herein, we produced TESs using serum-containing medium for the dermal component. The next step is thus to develop an entirely serum-free system for dermal and epidermal cell cultivation and TES production. The successful production of self-assembled stroma using dermal fibroblasts cultured in a serum-free and/or xenogen-free medium was recently reported [60], which we believe makes it likely that we will succeed. As for Surge SFM, it will need to be tested in parallel with other keratinocyte-specific serum-free commercially available media to confirm its benefits [24] but, all in all, this new serum-free medium shows great promise for basic and clinical research in the skin tissue engineering field.

## Data Availability

The data presented in this study are available on request from the corresponding author.

## Supplementary Materials

The following supporting information can be downloaded at: https://www.mdpi.com/article/10.3390/ijms24031821/s1.
