# Supplementary material for: A Newly Developed Chemically Defined Serum-Free Medium Suitable for Human Primary Keratinocyte Culture and Tissue Engineering Applications
Source: Int J Mol Sci. 2023 Jan 17;24(3):1821. doi: 10.3390/ijms24031821 (PMC9915451; doi:10.3390/ijms24031821)
Supplement: Supplementary file 1 [file ijms-24-01821-s001.zip › ijms-2102492-supplementary.pdf]

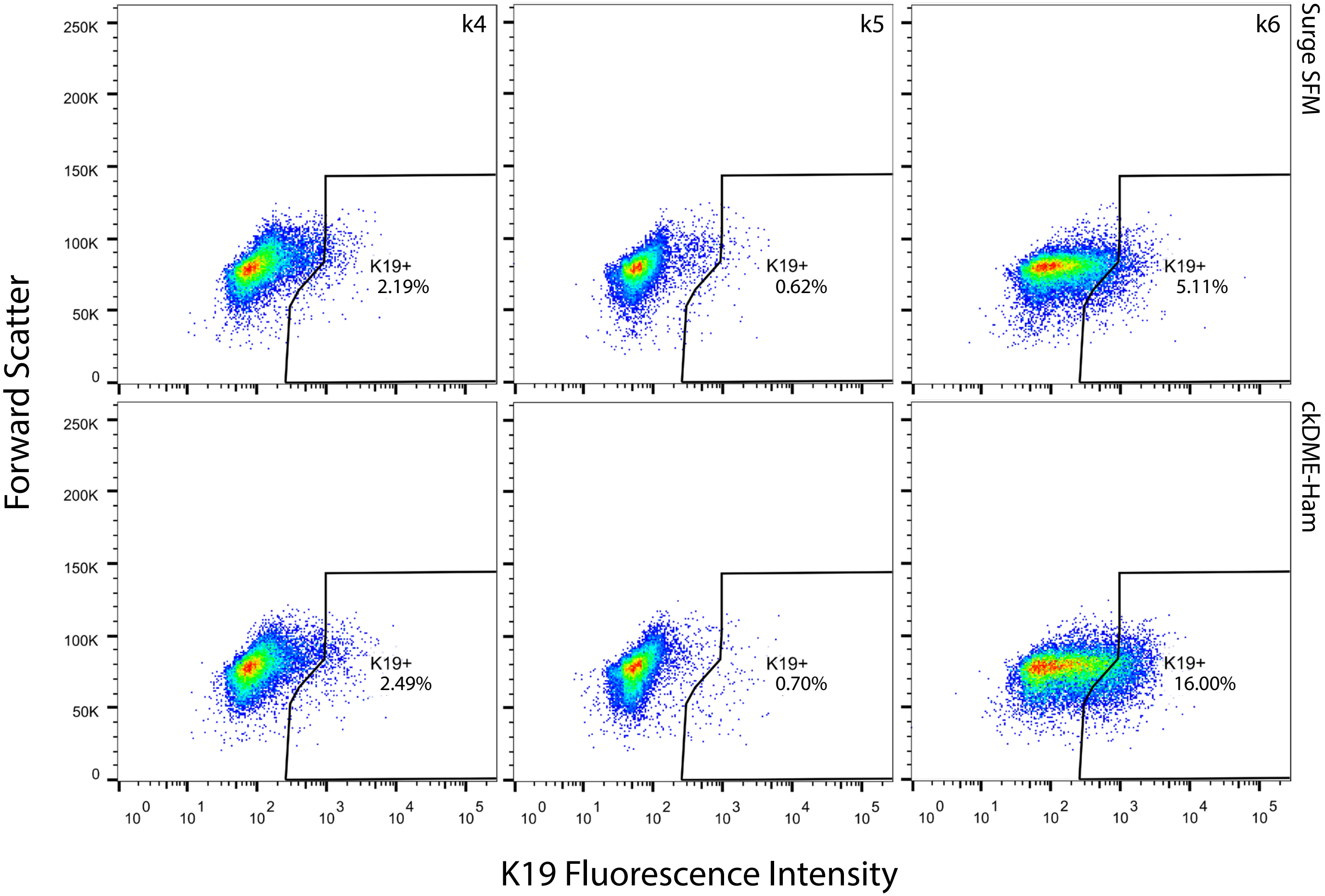

Figure S1. Representative example of flow cytometry data presented as dot plots of relative fluorescence intensity for K19 after immunolabelling with anti-human K19 and PE-conjugated secondary antibody. Presented here are populations k4, k5, and k6 in P3 cultured in either ckDME-Ham or Surge SFM.

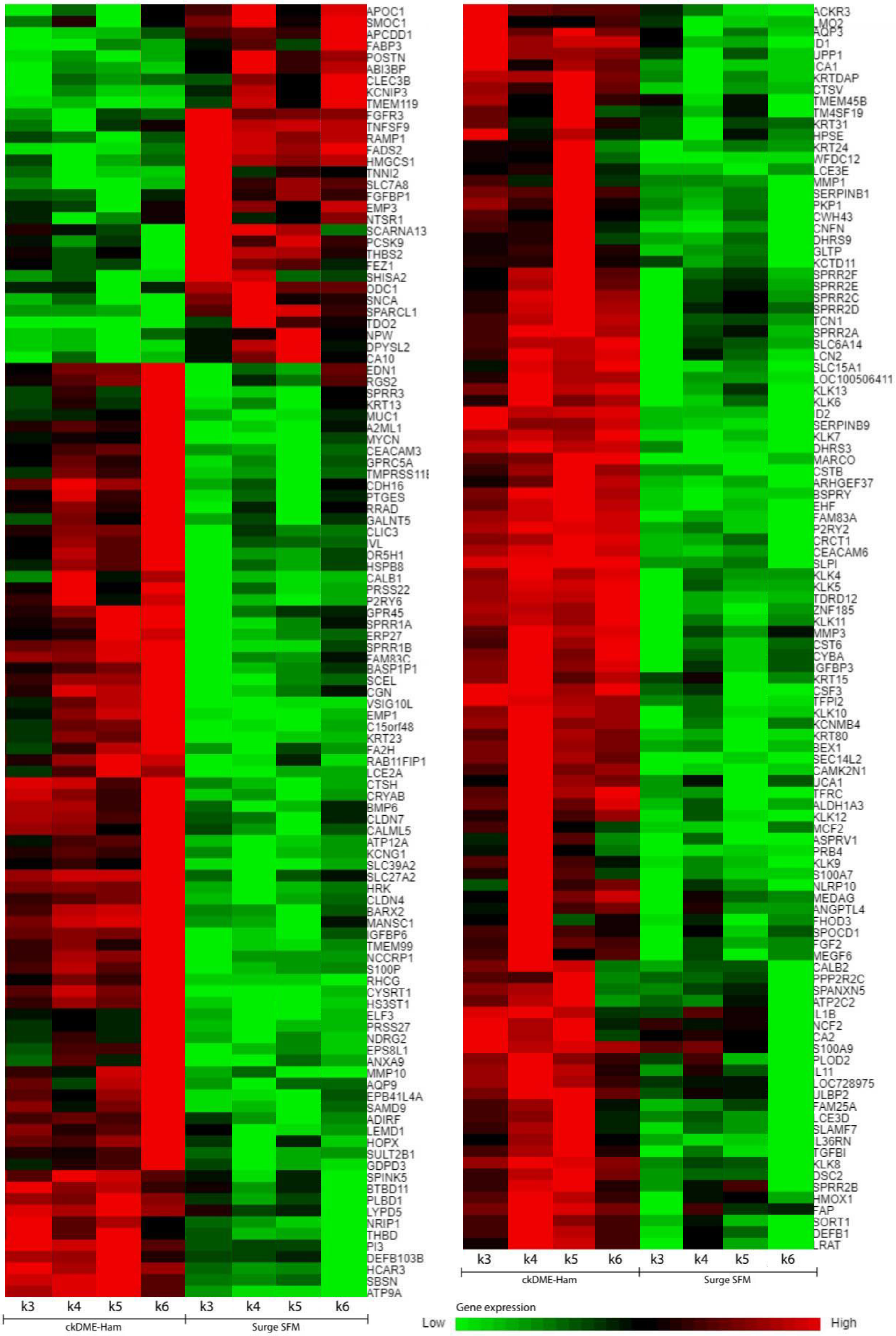

Figure S2. Heat map of all the statistically significantly differentially expressed genes between keratinocytes cultured with either Surge SFM or ckDME-Ham. All four populations (k3, k4, k5, k6) used for the microarray assay are presented.
